# *Mycobacterium tuberculosis* and *Mycobacterium avium* Complex Cutaneous Co-Infection: Diagnostic and Therapeutic Challenges

**DOI:** 10.3390/pathogens15070774

**Published:** 2026-07-22

**Authors:** Minhua Weng, Guizhong Zhou, Qiuping Wu, Qiong Chen, Jiabin Li, Zheng Wang, Wenting Li

**Affiliations:** 1 Department of Infectious and Tropical Diseases, The Second Affiliated Hospital of Hainan Medical University, Haikou 570311, China; wmh24301015@163.com (M.W.); zhouguizhong68@163.com (G.Z.); 15103662353@163.com (Q.W.); 17733168895@163.com (Q.C.); 2 Department of Internal Medicine, The Hainan Public Health Clinical Center, Haikou 571129, China; 3 Department of Infectious Diseases, The First Affiliated Hospital of Anhui Medical University, Hefei 230022, China; lijiabin@ahmu.edu.cn; 4 Department of Respiratory and Critical Care Medicine, Henan Provincial People’s Hospital, Zhengzhou 450003, China

**Keywords:** *Mycobacterium avium* complex, *Mycobacterium tuberculosis*, cutaneous coinfection, metagenomic next-generation sequencing

## Abstract

Cutaneous co-infection with *Mycobacterium tuberculosis* (MTB) and *Mycobacterium avium* complex (MAC) is extremely rare and easily missed due to overlapping histopathological features. We report a previously healthy, HIV-negative middle-aged woman who presented with a progressive destructive mass in the left inguinal-perineal region. Imaging revealed sinus tract formation, osteolytic bone lesions, and chronic inflammation in the right middle lobe of the lung. Initial metagenomic next-generation sequencing (mNGS) detected 3756 reads of the *Mycobacterium tuberculosis* complex (MTBC) and 111 reads of *Mycobacterium intracellulare* (*M. intracellulare*); the latter was interpreted as possible colonization or contamination because of its low abundance. Empirical anti-tuberculosis therapy produced only transient partial improvement, followed by paradoxical worsening, local recurrence, and new bone destruction. After a high suspicion of mixed infection, a MAC-directed combination regimen (including azithromycin and a short course of amikacin) was added, leading to complete clinical cure; subsequent repeat cultures confirmed the presence of MAC. This is the first report of cutaneous MTB-MAC co-infection in the inguinal-perineal region of an adult without overt immune abnormalities, accompanied by disseminated bone lesions. This case highlights that in regions where nontuberculous mycobacteria (NTM) are co-endemic, atypical destructive skin lesions with paradoxical worsening despite initial response to anti-tuberculosis therapy should raise suspicion of MAC co-infection. The combination of mNGS and conventional culture facilitates identification of mixed infections and guides precision therapy, but mNGS results must be interpreted cautiously in the clinical context.

## 1. Introduction

Cutaneous mycobacterial infections constitute a heterogeneous group of chronic infectious diseases caused by mycobacteria invading the skin and subcutaneous tissues. These infections are broadly classified into three categories: *Mycobacterium tuberculosis* complex (MTBC), nontuberculous mycobacteria (NTM), and *Mycobacterium leprae*. Although *Mycobacterium tuberculosis* (MTB) and NTM share histopathologic features such as granulomatous inflammation, their distinct epidemiologic profiles, routes of transmission, therapeutic responses, and prognoses make accurate differential diagnosis imperative [1].

Cutaneous tuberculosis (CTB) accounts for approximately 1~2% of all tuberculosis cases, predominantly observed in TB-endemic regions. It manifests through two primary pathways: exogenous inoculation, as seen in tuberculosis verrucosa cutis and tuberculous chancre, and endogenous dissemination, exemplified by lupus vulgaris and scrofuloderma [2,3]. The expanding population of immunocompromised hosts—particularly those with advanced HIV disease—has reshaped the epidemiologic landscape, with atypical presentations and disseminated disease becoming increasingly prevalent [4]. This transformation has substantially complicated timely recognition, as CTB mimics numerous dermatologic conditions, frequently resulting in diagnostic delays [5]. Compounding this challenge, the paucibacillary nature of cutaneous lesions often undermines the sensitivity of conventional diagnostic modalities such as acid-fast bacilli (AFB) staining and culture, necessitating molecular techniques like polymerase chain reaction (PCR) for definitive confirmation [6].

The *Mycobacterium avium* complex (MAC)—a ubiquitous group of slow-growing environmental NTM species—is the most frequently isolated NTM group in clinical practice. Primary cutaneous MAC infection remains exceptionally rare. In a large retrospective cohort of 142 patients with skin and soft-tissue NTM infections, MAC ranked as the second most common isolate; notably, 90.0% of patients with MAC had underlying immunocompromising conditions, and this subgroup demonstrated a predilection for disseminated rather than localized cutaneous disease. However, an accumulating body of case reports has documented primary cutaneous MAC infection in immunocompetent individuals with specific environmental exposures, including trauma, tattoos, and hot tub use, broadening the at-risk population beyond traditionally recognized groups. Unlike CTB, MAC exhibits intrinsic resistance to conventional first-line anti-tuberculous agents such as isoniazid and ethambutol, mandating clarithromycin or azithromycin-based macrolide-containing regimens. Accordingly, precise identification through culture and molecular methods (e.g., gene sequencing or matrix-assisted laser desorption/ionization time-of-flight mass spectrometry, MALDI-TOF MS) is critical for guiding appropriate antimicrobial therapy [7,8,9,10,11,12].

Simultaneous cutaneous co-infection by MTB and MAC is an extraordinarily uncommon event, with only isolated cases reported in the English-language literature [13,14]. Most documented instances have occurred in severely immunocompromised hosts, particularly those with advanced acquired immunodeficiency syndrome (AIDS), raising the possibility that profound immunodeficiency may create a permissive niche for the synchronous proliferation of phylogenetically distinct mycobacterial species within the same cutaneous microenvironment. Distinguishing true co-infection from colonization or sequential infection poses a formidable diagnostic challenge that remains poorly addressed by current guidelines [15]. Moreover, the diverging antimicrobial susceptibilities of MTB and MAC—combined with the potential for drug–drug interactions and overlapping toxicities—render therapeutic decision-making extraordinarily complex in these rare patients.

**Figure 1:**
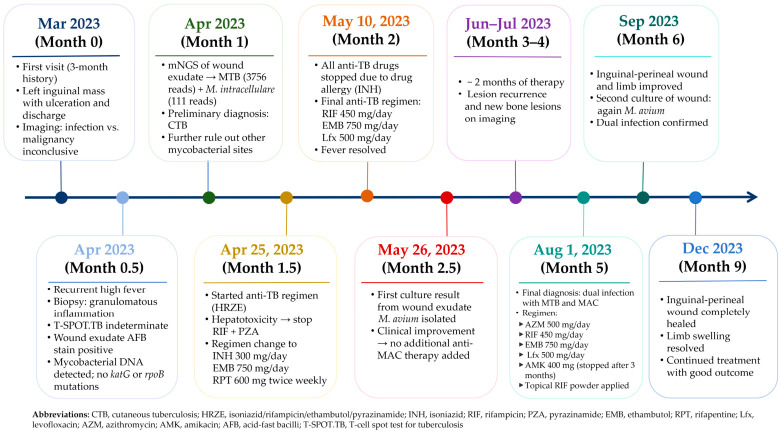
Diagnostic and treatment flowchart.

Herein, we report a case of cutaneous co-infection with MTB and MAC in a 49-year-old female patient, accompanied by disseminated osteolytic bone lesions and chronic pulmonary inflammation. This case illustrates the diagnostic pitfalls encountered when two phylogenetically distinct mycobacterial species concurrently infect the skin and highlights the therapeutic challenges inherent in managing dual mycobacterial co-infection (Figure 1).

## 2. Case Presentation

### 2.1. Clinical Findings

A 49-year-old woman from Hainan Province, a tropical region of China, working as a construction site laborer, presented to the Department of Traumatology with a three-month history of a progressively enlarging mass in the left inguinal region, accompanied by ulceration and purulent discharge.

The patient had been previously healthy. There was no history of tuberculosis (TB) or known contact with TB, no history of trauma, and no history of drug or food allergies. She denied smoking or alcohol use. There was no history of diabetes mellitus (DM) or other chronic diseases, and no history of exposure to hazardous chemicals or radiation. Family history and personal history were unremarkable.

### 2.2. Initial Evaluation

**Figure 2:**
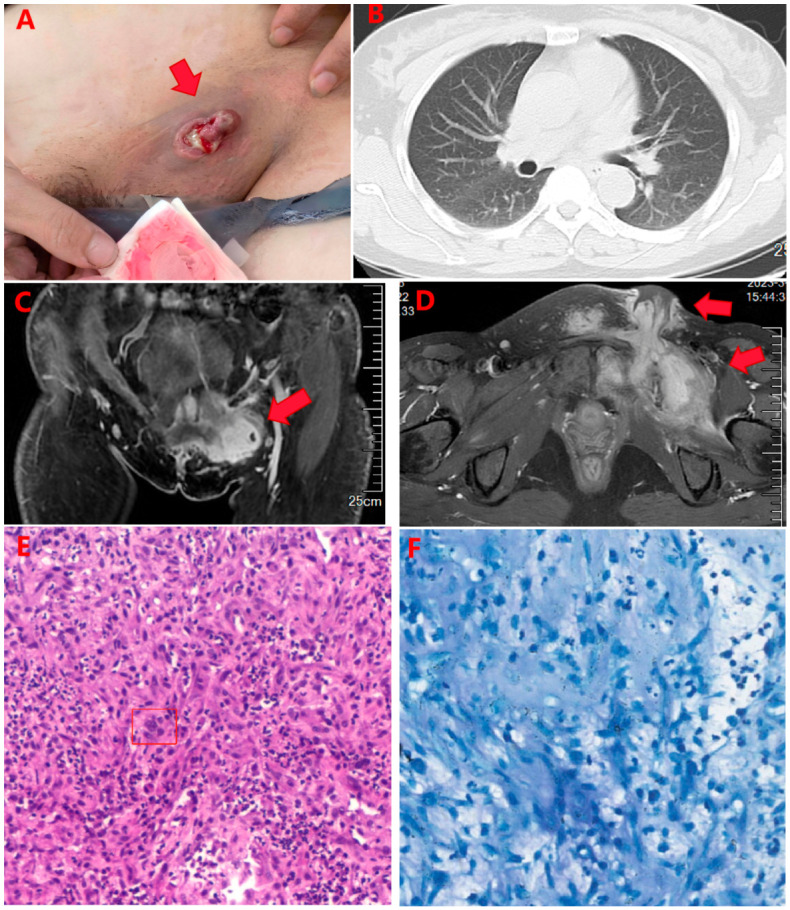
Clinical, radiological, and pathological findings at initial presentation. (**A**) Photograph of the left inguinal region showing a 4 cm × 4 cm raised soft-tissue mass with ill-defined borders and purulent discharge (red arrow). (**B**) Chest CT revealing mild chronic inflammation in the medial segment of the right middle lobe. (**C**) Pelvic contrast-enhanced MRI demonstrating multiple abnormal soft-tissue masses in the left inguinal and perineal regions with sinus tract formation (red arrow). (**D**) Contrast-enhanced MRI of the pelvis revealing extensive heterogeneous enhancement of the left pubis, perineal subcutaneous tissue, and obturator muscles, with poorly defined margins and a sinus tract in the left perineal region. (red arrow). (**E**) Histopathological examination of the perineal lesion showing granulation tissue with focal aggregation of multinucleated giant cells (red box), consistent with granulomatous inflammation (hematoxylin and eosin stain; H&E). (**F**) Acid-fast staining (Ziehl-Neelsen) of the biopsy specimen is negative for AFB.

On admission, physical examination revealed a raised mass measuring approximately 4 cm × 4 cm in the left inguinal area, extending to the muscular layer. The mass was soft, non-mobile, with reddish-white purulent drainage, and the distal border was ill-defined (Figure 2A). No other abnormalities were found on physical examination.

Chest computed tomography (CT) revealed minimal chronic inflammation in the medial segment of the right middle lobe (Figure 2B). Pelvic magnetic resonance imaging (MRI) demonstrated multiple soft-tissue masses with abnormal signal intensity in the left inguinal and perineal regions, accompanied by a sinus tract in the left perineum. The lesions exhibited invasive growth involving adjacent and distant soft tissues, together with osteolytic destruction of the left pubic and ischial bones (Figure 2C,D). Initial imaging assessment could not clearly distinguish between infectious disease and malignancy.

Laboratory findings (Table 1) showed an elevated white blood cell count of 11.72 × 10^9^/L, a red blood cell count of 4.09 × 10^12^/L, hemoglobin of 102 g/L (mild anemia), and thrombocytosis (platelets 452 × 10^9^/L). C-reactive protein (CRP) was markedly elevated to 23.5 mg/L, while procalcitonin (PCT) was at the upper limit of normal (0.1 ng/mL). The erythrocyte sedimentation rate (ESR) was significantly increased to 80 mm/h. Albumin was slightly decreased (39.9 g/L), and alanine aminotransferase (ALT) was marginally below the lower reference limit (6 U/L). Other liver function tests (AST, total bilirubin), renal function, and glucose were within normal ranges. Ferritin (161 ng/mL), rheumatoid factor (6 IU/mL), and HLA-B27 were all negative/normal. Tests for SARS-CoV-2, influenza A and B viruses, HIV, and various tumor markers were negative.

### 2.3. Initial Diagnostic Workup and Suspicion

During hospitalization, the patient developed recurrent high fever (peak 40 °C) that responded poorly to piperacillin-tazobactam. Percutaneous needle biopsy of the inguinal-perineal region showed granulomatous inflammation (Figure 2E, boxed area), but staining for AFB was negative (Figure 2F). Multidisciplinary discussion revealed that acid-fast staining of wound exudate detected scattered acid-fast bacilli, while the T-cell Spot Test (T-SPOT.TB) result was indeterminate. A drug resistance gene chip test for MTB (isoniazid and rifampicin) confirmed the presence of mycobacterial DNA in the specimen, with no mutations in the *katG* or *rpoB* genes associated with drug resistance. Based on these findings, the patient was transferred to the Department of Infectious Diseases on 19 April 2023, for planned initiation of targeted anti-mycobacterial therapy.

The patient’s persistent anemia showed no definite pathological changes on bone marrow aspiration and biopsy, and was attributed to anemia of chronic disease secondary to chronic mycobacterial infection. Chest imaging revealed minimal chronic inflammation in the medial segment of the right middle lobe. Although the patient had no cough or sputum production during the disease course, bronchoscopy, lymphocyte subset analysis and autoantibody screening were recommended to further characterize the pulmonary findings and assess immune status—all of which the patient declined. Given the diagnostic value of the interferon-gamma release assay (IGRA) for mycobacterial infection, a repeat T-SPOT.TB still yielded an indeterminate result. During hospitalization, three sputum smears for AFB were negative. On 23 April 2023, an inguinal-perineal wound exudate sample was sent to Hangzhou Shengting Medical Technology Co., Ltd., Hangzhou, China, for metagenomic next-generation sequencing (mNGS), which detected 3756 specific reads of MTBC and 111 reads of *Mycobacterium intracellulare* (*M. intracellulare*).

Given that *M. intracellulare* is a common environmental saprophyte, its detection did not exclude the possibility of specimen contamination—especially considering that this was a single wound exudate sample with few sequence reads (111 reads vs. 3756 for MTBC), making it difficult to distinguish true infection from colonization or contamination. According to the recommendation by Miller et al., the clinical relevance of environmentally ubiquitous opportunistic pathogens (such as MAC) should be interpreted with caution in the absence of negative controls or when clinical suspicion is not strong [16,17]. Moreover, the patient had repeatedly negative sputum smears, no typical radiological features of pulmonary tuberculosis (PTB), and conventional culture and species identification of the pathogen results from both sputum and wound exudate required approximately two months to become available. Taken together, the initial clinical diagnosis was cutaneous mycobacteria infection. Concurrent pulmonary or systemic mycobacteria infection was inadequate to be diagnosed, if not completely excluded, according to the clinical and laboratory data.

### 2.4. Adverse Drug Reactions and Regimen Adjustments

Shortly after treatment initiation, the patient developed liver injury (elevated ALT/AST). Among first-line anti-tuberculosis drugs, isoniazid, rifampicin and pyrazinamide all carry known hepatotoxic risks, which increase when used in combination. Rifampicin and pyrazinamide were considered the most likely causative agents; they were discontinued and hepatoprotective drugs were introduced. The regimen was empirically adjusted to isoniazid 300 mg once daily, ethambutol 750 mg once daily, and rifapentine 600 mg twice weekly. Thereafter, liver function gradually improved.

However, on 10 May 2023, the patient developed erythematous maculopapular rash on the trunk and extremities, accompanied by pruritus. Dermatology consultation attributed the rash to an anti-tuberculosis drug allergy. All first-line anti-tuberculosis drugs were discontinued, and the patient was treated with methylprednisolone combined with cetirizine, after which the rash completely resolved. Subsequent sequential drug challenge tests (isoniazid, rifampicin, ethambutol, levofloxacin) under close monitoring revealed that re-administration of isoniazid reproduced generalized pruritus and rash (positive challenge), confirming an allergic reaction to isoniazid.

The final anti-tuberculosis regimen was adjusted to rifampicin 450 mg once daily, ethambutol 750 mg once daily, and levofloxacin 500 mg once daily. After this adjustment, the patient’s systemic and local symptoms improved, body temperature normalized, and no new liver injury occurred. She was followed up regularly in the outpatient clinic.

### 2.5. First Culture Result and Clinical Decision

On 26 May 2023, the first conventional mycobacterial culture of wound exudate from the inguinal-perineal region returned positive for *Mycobacterium avium* (*M. avium*); however, drug susceptibility testing could not be performed owing to laboratory limitations. The subsequent, sputum culture for MTB or MAC was negative. At that time, the left inguinal-perineal wound had already shown a tendency toward healing, with decreasing discharge. According to the ATS/ERS/ESCMID/IDSA 2020 clinical practice guideline for the treatment of nontuberculous mycobacterial pulmonary disease [17], a single NTM isolate from a site prone to contamination (e.g., skin and soft tissue) should be interpreted with caution, as it may represent environmental colonization or specimen contamination rather than definitive evidence of active infection. Although the guideline primarily addresses pulmonary disease, its principle of cautious interpretation of NTM isolates can reasonably be extrapolated to extrapulmonary specimens.

Moreover, the patient had already experienced serious adverse drug reactions (ADRs) during earlier treatment, including drug allergy (isoniazid) and hepatotoxicity (rifampicin + pyrazinamide). Initiating specific therapy for a pathogen (MAC) of uncertain pathogenicity at this stage might have increased the patient’s treatment burden, interfered with the already partially effective regimen, and complicated attribution of adverse reactions.

### 2.6. Disease Progression and Final Diagnosis

**Figure 3:**
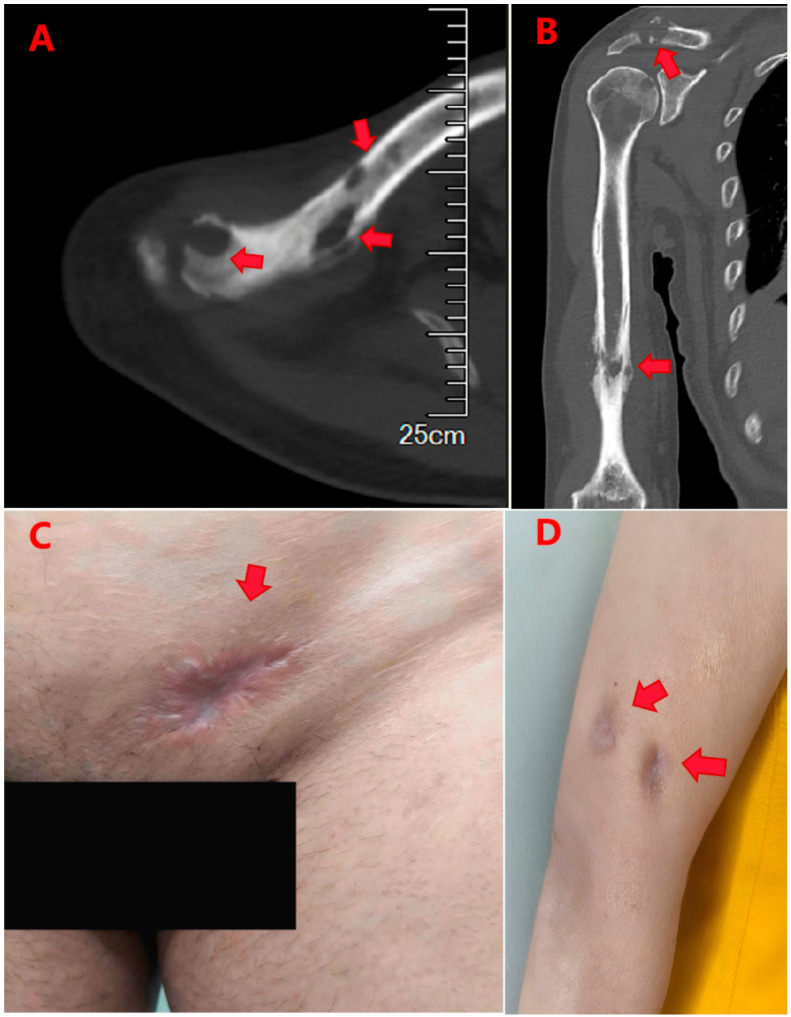
Disease progression and treatment response during follow-up. (**A**) Chest CT showing multiple osteolytic lesions in the right clavicle (red arrows). (**B**) Chest CT demonstrating osteolytic lesions in the right clavicle and distal humerus (red arrows). (**C**) Photograph of the left inguinal region showing complete wound healing following combination therapy (red arrow). (**D**) Photograph of the right upper extremity showing resolution of swelling and complete healing after continued treatment (red arrow).

After approximately two months of regular anti-tuberculosis treatment (July 2023), the patient developed local recurrence of the left inguinal lesion and new-onset swelling of the right upper limb. CT revealed multifocal osteolytic lesions involving the bilateral clavicles, right humerus, scapula, left 7th rib, and the 11th thoracic vertebra (Figure 3A,B, red arrows). Chest CT scan showed minimal bilateral chronic infiltration with no significant changes from previous imaging series. Whole body positron emission tomography–computed tomography (PET-CT) or positron emission tomography–magnetic resonance imaging (PET-MRI) scan was suggested, albeit not performed owing to the patient’s financial constraints.

Integrating the previous mNGS result (3756 MTBC reads and 111 *M. intracellulare* reads), serial microbiological culture results, and the suboptimal response to initial anti-tuberculosis therapy, a final diagnosis of dual infection with MTB and MAC was established. Accordingly, on 1 August 2023, the treatment regimen was changed to combination therapy covering both pathogens: azithromycin 500 mg once daily, rifampicin 450 mg once daily, ethambutol 750 mg once daily, levofloxacin 500 mg once daily, and amikacin 400 mg once daily (discontinued after three months of treatment). In addition, rifampicin powder (from capsules) was applied topically to the left inguinal lesion as local adjunctive therapy. Another specimen of inguinal-perineal discharge was collected for mycobacterial culture to guide subsequent management. After the regimen adjustment, the inguinal-perineal wound gradually healed, and the right upper limb swelling markedly improved.

On 15 September 2023, a second independent culture of inguinal-perineal wound discharge again isolated *M. avium*—two independent positive cultures with identical findings further supported the diagnosis of dual infection with MAC and definitively ruled out single-specimen contamination. After four months of the combination therapy (December 2023), the inguinal-perineal wound was completely healed (Figure 3C, red arrow).

During later follow-up, the patient experienced a transient episode of exacerbated right upper limb swelling with mild ulceration, but without fever, joint pain, or limitation of movement. This was interpreted as spontaneous rupture and drainage of deep caseous necrotic material to the body surface during treatment, a transient and self-limited manifestation in the healing process of the lesion. With continued treatment, the swelling progressively resolved and the lesion eventually healed (Figure 3D). The patient has remained stable with no signs of recurrence to date.

## 3. Discussion

### 3.1. Clinical Challenges in This Case and MTB-MAC Cutaneous Co-Infection

We report here a case of MTB-MAC cutaneous co-infection, which poses a rare entity and diagnostic challenge. Since both organisms produce highly similar granulomatous inflammation clinically and histopathologically, conventional microbiological methods (such as AFB and mycobacterial culture) are not only difficult to differentiate but also have long turnaround times. Moreover, differences in growth characteristics between the two pathogens, or selective suppression by prior anti-tuberculosis drugs, can easily lead to under-detection of one organism during culture. In this context, mNGS—a culture-independent technique that enables unbiased detection of microbial nucleic acids—provides a crucial and fast tool for identifying such mixed infections. In our case, the multidisciplinary diagnosis could be made when combining a high mNGS copy number with the clinical picture. The diagnosis based on mNGS results was further supported by her disease course and treatment responses. It is important to emphasize that the core pathological basis of this case was cutaneous co-infection with MTB and MAC, and clinical management was directed primarily at the skin and soft-tissue lesions, rather than being driven by an initial diagnostic focus on active pulmonary tuberculosis. The final diagnosis was established not through active screening for pulmonary tuberculosis, but through the integrated application of skin biopsy histopathology, mNGS, and culture of wound exudate, which together enabled accurate identification of the mixed infection. In addition, the detection of *M. intracellulare* by mNGS and the identification of *M. avium* by culture corroborate each other, together confirming MAC infection; the discrepancy between the two methods reflects only a methodological difference in species-level identification, without affecting either the clinical diagnosis or the therapeutic approach.

Pulmonary MTB infection often precedes or complicates with cutaneous infections. So frequently that sometimes this may offer a diagnostic clue for extrapulmonary infection. In our case, however, clues of pulmonary infection are too insufficient to make a diagnosis. She presented with no significant respiratory symptoms on admission. Chest CT revealed only minimal chronic inflammation in the medial segment of the right middle lobe, without the typical tree-in-bud opacities, cavitation, or consolidation pattern of active pulmonary tuberculosis. Although these imaging features were non-specific, the location of the lesion prompted further consideration. The right middle lobe has an anatomically narrow orifice, making it susceptible to obstruction and chronic inflammation from various etiologies, including endobronchial tuberculosis (EBTB). EBTB can present as right middle lobe syndrome (RMLS)—a chronic or recurrent collapse of the right middle lobe—with non-specific imaging findings and negative sputum smears. In a series of 22 patients with EBTB presenting as RMLS, cough with sputum was the most common manifestation, but two patients were entirely asymptomatic. Furthermore, the reported incidence of tuberculosis as a cause of RMLS ranges from 9% to 26.1% in various case series from Asian countries [18,19,20]. Thus, the mild chronic inflammation in the right middle lobe of our patient, while lacking classic imaging features of active pulmonary tuberculosis, cannot exclude the possibility of underlying EBTB or prior subclinical tuberculous involvement. Follow-up CT showed no appreciable evolution or resolution of this lesion. In the absence of higher-quality specimens such as bronchoalveolar lavage fluid, sputum PCR testing would still have offered important supplementary value for identifying pulmonary pathogens. During the clinical course, we intended to perform bronchoalveolar lavage for pathogen testing or sputum PCR; however, neither could be completed due to patient-related factors.

Whole body functional imaging (i.e., PET-CT or PET-MRI scan) has been used in the diagnosis and evaluation of mycobacteria infection. These scans are widely used in clinical settings, which allows for systemic identification of occult lesions, including lymph nodes, liver, spleen, bone or bone marrow, subcutaneous region and muscle. The spectra of involving organs or tissues may provide diagnostic or differential information. In addition, the glucose uptake status of the lesions, in combination with conventional CT or MRI images, may give further diagnostic clues and facilitate the differential diagnosis of mycobacteria from abscesses, IgG4-related diseases, histiocytosis or malignancies. Previous studies have reported a moderate-to-high value of PET scans in diagnosing tuberculosis. If needed, PET-CT might be performed to fully assess the extent of systemic involvement, as well as to characterize the full disease burden. Systemic lesions and higher glucose uptake is suggested to reflect the disease activity and may guide the choice of therapy. However, limited data has directly compared the PET manifestations of tuberculosis or NTM, tuberculosis and NTM co-infection, or infection with different species of NTM [21,22]. Therefore, differential diagnosis between species or co-infection could hardly be made by PET scan considering the complexity of our current case.

Even after diagnosis is established, management of mixed mycobacterial infections remains therapeutically challenging. In the present case, treatment was complicated by severe adverse reactions, including hepatotoxicity and isoniazid allergy. The patient ultimately achieved clinical cure with a macrolide-containing combination regimen (azithromycin, rifampicin, ethambutol, levofloxacin, and amikacin). This experience underscores the need to carefully balance drug susceptibility, tolerability, and potential interactions when designing regimens for dual mycobacterial infections. Furthermore, topical rifampicin powder was used as an adjunctive treatment; its pharmacokinetics and clinical efficacy have not been systematically validated, and this report merely describes an empirical attempt. Finally, a single case cannot fully rule out region-specific environmental exposure factors (the patient came from Hainan, a tropical region of China, and worked as a construction site laborer). Further validation with more cases is needed.

### 3.2. Possible Explanations of Co-Infection and Refractoriness

Beyond the diagnostic and therapeutic complexities discussed above, the underlying mechanisms that allow dual infection with MTB and MAC in the same host might be intrinsically complex, while cutaneous co-infection is even rarer. NTM and uncommon co-infections with NTM are well documented in immunocompromised patients. Initial assessment classified the patient as immunocompetent based on routine criteria (HIV-negative, no known immunosuppression). However, subsequent clinical course and laboratory parameters suggest that a more nuanced re-evaluation of her immune status may be warranted. Clinically, the patient presented with recurrent high fever (peak 40 °C) unresponsive to broad-spectrum antibiotics, persistent anemia (lowest hemoglobin 102 g/L), reactive thrombocytosis (452 × 10^9^/L), evidence of chronic inflammation (mildly decreased albumin 39.9 g/L), and an indeterminate IGRA result—the latter being relatively uncommon in immunocompetent individuals but more frequently observed in patients with chronic wasting conditions, malnutrition, or occult immune dysfunction [23].

Ideally, for patients with suspected mycobacterial co-infection—particularly when the clinical picture suggests a chronic inflammatory wasting state—routine evaluation should include peripheral blood lymphocyte subsets (CD3^+^, CD4^+^, CD8^+^ T-cell counts and ratios), serum immunoglobulin quantification (IgG, IgA, IgM), and assessment of specific antibody responses. Moreover, given the patient’s recurrent infections and negative HIV status, two additional tests warrant consideration. Whole-exome sequencing (WES) can be used to screen for pathogenic variants associated with Mendelian susceptibility to mycobacterial diseases (MSMD), a group of inborn errors of immunity caused by defects in IL-12/IFN-γ pathway genes (including IFNGR1, IFNGR2, STAT1, IL12B, IL12RB1, ISG15, IRF8, NEMO and CYBB) that predispose to severe infections caused by weakly virulent mycobacteria without overt abnormalities in routine immunological parameters [24]. WES represents one of the most effective tools for identifying MSMD genotypes, and MSMD patients may present with either localized or disseminated disease following infection with environmental mycobacteria [25]. Anti-interferon-γ autoantibody (AIGA) testing helps identify another non-HIV-related adult-onset immunodeficiency (AOID). AIGA syndrome is a rare acquired immunodeficiency strongly associated with diverse opportunistic infections, most commonly nontuberculous mycobacteria (NTM, 55.69%), followed by Talaromyces marneffei (26.98%), Salmonella spp. (12.43%), MTB (9.34%) and varicella-zoster virus (9.57%). Notably, skin involvement is one of the most common multi-organ manifestations (45.16%), often presenting as nodules, erythema, ulcers and other cutaneous lesions [26,27,28]. These tests help identify potential underlying conditions such as MSMD, AIGA syndrome, common variable immunodeficiency (CVID) or idiopathic CD4^+^ lymphocytopenia (ICL), which may predispose the host to environmental MAC infection. A comprehensive examination of the immune status, although declined by our patient, may reveal the underlying congenital or adaptive immunodeficiency, such as MSMD, AIGA syndrome, CVID, or ICL, which may predispose the co-infections. The examination list may include lymphocyte subset analysis, immunoglobulin subset assay, autoantibody screening, WES or whole genome sequencing (WGS), AIGA testing, and so on. WES or WGS may also reveal certain genetic polymorphisms or mutation loci, which have been suggested to be the genetic predispositions of co-infections by previous studies [29,30].

Of note, although bone marrow aspiration excluded a primary marrow disorder in our patient, the combination of normocytic normochromic anemia and thrombocytosis strongly fits the diagnostic framework of anemia of chronic disease (ACD). ACD is typically driven by persistent infection, autoimmune disease, or malignancy, and depends on inflammatory cytokines (e.g., IL-6, TNF-α, IFN-γ) released by immune effector cells—particularly T lymphocytes and monocytes—which suppress erythroid hematopoiesis and iron metabolism [31]. Thus, the patient’s chronic mycobacterial burden itself constitutes a sustained state of immune activation. This persistent immune activation may represent both a compensatory mechanism for host control of infection and, over a prolonged course, a consumption of immune reserves that leads to a degree of functional immune deficiency [32].

Another possible explanation is the secondary hit theory. Post-TB structural lung damage, such as post-tuberculous pulmonary fibrosis or apical scarring, can itself create a permissive microenvironment for secondary colonization or infection by NTM, potentially leading to mixed MTB-NTM infection even in immunocompetent hosts [33]. In our patient, the mild chronic inflammation in the medial segment of the right middle lobe on chest CT (Figure 2B), while not characteristic of active tuberculosis, may nonetheless indicate an underlying pulmonary structural abnormality or chronic inflammatory milieu. Notably, primary cutaneous mycobacterial infection without pulmonary involvement has also been reported in the literature [34,35].

In the present case, the initial HRZE regimen contained ethambutol (partially active against MAC), which may have partially suppressed MAC growth. This explains the initial wound healing observed after anti-tuberculosis therapy, which paradoxically masked the pathogenic role of MAC and ultimately led to recurrence and bone dissemination. The empirical regimen directed against MTB may, on one hand, create a selective ecological niche for MAC—an organism intrinsically resistant to several first-line drugs—and, on the other hand, impair host immune surveillance, thereby promoting or exacerbating MAC infection. Pahuja et al. lent immunological support to this hypothesis, showing that anti-tuberculosis therapy can impair host immune control over mycobacteria, thereby increasing the risk of relapse or co-infection [36]. This clinical trajectory serves as a reminder that even when initial anti-tuberculosis therapy produces some clinical improvement, the possibility of mixed infection should not be readily dismissed in patients with cutaneous or soft-tissue MAC infection who are otherwise immunocompetent.

### 3.3. Literature Review of Similar Cases

To position the present case within the published literature, we systematically reviewed reported cases of dual MTB and MAC infection (Table 2). The PubMed, Web of Science and Google Scholar databases were searched up to March 2026. Inclusion criteria were English-language reports in which co-infection with MTB and MAC in the same patient was confirmed by culture, molecular diagnosis or histopathology. Reports of simple colonization, single-species infection and review articles were excluded. A total of 13 cases with relatively complete clinical data were identified.

In immunocompetent individuals (classified as “immunocompetent” or “minimally abnormal”), dual infection most often presents as localized disease confined to lymph nodes or the lungs, and typically requires molecular testing or surgical resection specimens to reveal the co-infection. In 2000, Ganesan et al. described a two-year-old girl who presented with cervical lymphadenopathy; initially only drug-resistant MAC was isolated, but surgical excision of the lymph node later yielded fully susceptible MTB, highlighting the diagnostic value of combined culture of resected tissue [37]. In 2008, Tuerlinckx et al. [38] reported a 7.5-year-old girl adopted from India who presented with intrathoracic lymphadenopathy and a strongly positive tuberculin skin test, but gastric aspirate and bronchial washings were culture-negative. Mediastinoscopic lymph node biopsy with the INNO Line Probe Assay (INNO-LiPA) reverse hybridization simultaneously detected MTBC and MAC from the culture, whereas conventional culture grew only MAC. The child recovered completely after nine months of treatment with clarithromycin, isoniazid and rifampicin. This case underscores the critical role of molecular diagnostics in differentiating extrapulmonary tuberculosis in children, especially when culture grows only the dominant species. In 2004, Damian et al. reported a 22-year-old immunocompetent woman with pulmonary co-infection of MTB and MAC. Her condition failed to improve on four-drug (HRZE) anti-tuberculosis therapy, but adding a clarithromycin-containing anti-MAC regimen led to clinical improvement, suggesting that co-infection should be suspected when the response to anti-tuberculosis therapy is suboptimal [39]. In 2024, Karki et al. further described MTB-MAC co-infection in an immunocompetent patient, highlighting the key clinical challenge of distinguishing MAC colonization from true pathogenicity [33]. In 2026, Aryal et al. reported a 48-year-old immunocompetent man who presented with cough, fever and weight loss; imaging showed thick-walled cavities in both upper lobes. Sputum culture simultaneously grew MTB, *Mycobacterium abscessus* (*M. abscessus*) and MAC; the *M. abscessus* isolate was susceptible to amikacin but intermediate to cefoxitin and imipenem. Despite the addition of amikacin, cefoxitin and azithromycin to a four-drug anti-tuberculosis regimen, sputum smears remained positive for four months, illustrating the complexity of assessing treatment response in patients with multiple concurrent mycobacterial infections [40]. Together, these cases indicate that even in immunocompetent hosts, co-infection with MTB and MAC can occur, and its clinical presentation is often masked by a single dominant pathogen.

In the specific subgroup of cutaneous MTB-MAC co-infection, only isolated cases have been reported, and almost all have occurred in severely immunocompromised hosts. In 1990, Lombardo et al. reported the first case in an AIDS patient from whose skin lesions both MTB and MAC were isolated, opening the literature on this rare clinical phenomenon [13]. In 1997, Núñez et al. described an HIV-infected patient with concurrent cutaneous infection with cytomegalovirus, MTB and MAC, further expanding the pathogen spectrum of opportunistic skin infections in immunocompromised hosts [14]. The uniqueness of the present case lies in the fact that the patient was HIV-negative, had no obvious history of immunosuppression, presented predominantly with inguinal-perineal skin and soft-tissue involvement, and had disseminated bone lesions.

In immunocompromised populations—particularly patients with advanced HIV infection (CD4 count often <100/μL) or those on long-term glucocorticoid therapy—dual infection takes on a more complex and life-threatening disseminated pattern. In 2017, Sharma et al. using autopsy tissue and multiplex PCR, simultaneously detected MTB and MAC in the lung and lymph nodes of a chronic obstructive pulmonary disease (COPD) patient on long-term steroids, proposing that in an immunocompromised setting MAC may act as a synergistic pathogen rather than a mere colonizer [41]. The picture is even more severe in HIV-infected individuals: Sharma et al. (2015) rapidly diagnosed a deteriorating AIDS patient using multiplex PCR on lymph node aspirate; timely addition of clarithromycin led to marked improvement [42]. John et al. (2022) reported a female patient with a CD4 count of only 1 cell/μL who had a triple infection of MTB, disseminated MAC and parvovirus B19, illustrating the additive effect of multiple opportunistic infections under extreme immunosuppression [43]. In addition, Sharma et al. (2012) reported two AIDS patients with meningitis in whom cerebrospinal fluid multiplex PCR detected MTB-MAC co-infection; both died rapidly, highlighting the high lethality of central nervous system involvement [44]. Most recently, Lopez et al. [45] described a 45-year-old newly diagnosed HIV-positive man with a very low CD4 count who presented with fever, abdominal pain, generalized lymphadenopathy and hepatosplenomegaly. Imaging showed tree-in-bud opacities, pleural effusion and retroperitoneal lymphadenopathy. Because he could not tolerate rifampicin due to persistently elevated liver enzymes, an individualized regimen of isoniazid, ethambutol, azithromycin and rifabutin was used, and antiretroviral therapy was deferred to reduce the risk of immune reconstitution inflammatory syndrome (IRIS). This case underscores the complex therapeutic impact of hepatotoxicity, drug–drug interactions and IRIS risk when disseminated extrapulmonary TB co-occurs with pulmonary MAC infection in the setting of HIV-related immunosuppression.

## 4. Conclusions

Cutaneous co-infection with MTB and MAC is an extremely rare clinical entity. We report such a case in a 49-year-old female construction worker from Hainan, China. Although the patient was HIV-negative and initially classified as immunocompetent, her clinical presentation—recurrent fever, chronic anemia, elevated inflammatory markers, and an indeterminate IGRA—prompted a more nuanced re-evaluation of her immune status.

Diagnostically, the case was challenging: initial mNGS of wound exudate revealed high-abundance MTB reads but low-abundance MAC reads; the latter was interpreted as possible colonization given its low abundance in a single wound specimen. Empirical anti-tuberculosis therapy produced transient wound improvement, yet this paradoxical response was followed by local recurrence and new multifocal bone destruction—a trajectory that ultimately led to the diagnosis of dual infection, confirmed by mNGS, serial cultures, and imaging. Treatment was further complicated by severe adverse reactions, including hepatotoxicity and isoniazid allergy, before the patient achieved clinical cure with a macrolide-containing combination regimen (azithromycin, rifampicin, ethambutol, levofloxacin, and amikacin).

This case offers four actionable lessons for clinicians. First, HIV negativity should not be interpreted as immunocompetence. In patients presenting with chronic inflammatory wasting states and indeterminate IGRA results, a comprehensive immune workup—including lymphocyte subsets, immunoglobulins, and, when clinically indicated, WES and AIGA testing—should be considered. Second, low-abundance NTM reads on mNGS should not be automatically dismissed as contamination; when clinical suspicion persists, repeat sampling and serial cultures are essential to clarify their pathogenic role. Third, whole-body imaging such as PET-CT, though not performed in this case, can be valuable for assessing the extent of systemic involvement and ruling out occult malignancy—particularly in patients with invasive bone destruction and persistent inflammation, where imaging findings may help guide the diagnostic workup and inform treatment decisions. Fourth, initial improvement on anti-tuberculosis therapy does not rule out mixed infection—paradoxical worsening should prompt active investigation for NTM co-pathogens and consideration of a macrolide-containing regimen. Importantly, the subtle chronic pulmonary inflammation observed in this case—though non-specific on imaging—should not be viewed as an incidental bystander; it may represent a structural ‘second hit’ that creates a permissive niche for secondary NTM infection, underscoring the necessity of interpreting cutaneous mycobacterial disease within the broader pulmonary and immunological context of the host. The integrated use of mNGS and conventional culture is essential for accurate identification of mixed mycobacterial infections and for guiding targeted therapy.

## Figures and Tables

**Table 1:** Blood laboratory findings on admission.

Laboratory Test	Results	Reference Value
WBC	11.72 × 10^9^/L	3.5–9.5 × 10^9^/L
RBC	4.09 × 10^12^/L	3.8–5.1 × 10^12^/L
HGB	102 g/L	115–150 g/L
PLT	452 × 10^9^/L	125–350 × 10^9^/L
CRP	23.5 mg/L	0–6 mg/L
PCT	0.1 ng/mL	<0.1 ng/mL
ALT	6 U/L	7–40 U/L
AST	13 U/L	13–35 U/L
TBil	4.8 μmol/L	0–23 μmol/L
Albumin	39.9 g/L	40–55 g/L
Creatinine	67 μmol/L	41–73 μmol/L
Glucose	4.1 mmol/L	3.9–6.1 mmol/L
ESR	80 mm/h	0–20 mm/h
Ferritin	161 ng/mL	13–232 ng/mL
Rheumatoid factor	6 IU/mL	0–20 IU/mL
HLA-B27	Negative	Negative

**Table 2:** Summary of reported cases of MTB and MAC co-infection.

Case	Author, Year	Age/Sex	Immune Status (HIV/CD4/Other)	Site of Infection	Diagnostic Method	Treatment	Outcome
1	Lombardo et al., 1990 [13]	36/M	HIV (CD4 NR)	Skin	Culture	Multi-agent chemotherapy	Improved
2	Núñez et al., 1997 [14]	25/M	HIV (CD4 NR)/CMV	Skin	Culture/ histopathology	NR	NR
3	Karki et al., 2024 [33]	22/F	Immunocompetent	Lung	Culture/ molecular	ATT (HRZE)/AZM allergy	NR
4	Ganesan et al., 2000 [37]	2/F	Immunocompetent	Cervical lymph node	Culture (post-excision)	surgical excision/ATT (INH/RIF/EMB)	Recovered
5	Tuerlinckx et al., 2008 [38]	7.5/F	Immunocompetent	Intrathoracic lymph node	INNO-LiPA/ culture	ATT (NR)/CLR	Recovered
6	Damian et al., 2004 [39]	22/F	Immunocompetent	Lung	Culture	ATT failed, then added anti-MAC	Improved
7	Aryal et al., 2026 [40]	48/M	Immunocompetent	Lung	Sputum culture	ATT (HRZE)/AMK/ CFX/AZM	NR
8	Sharma et al., 2017 [41]	65/M	COPD on long-term steroids	Lung/ lymph node	Multiplex PCR	NR	Died
9	Sharma et al., 2015 [42]	35/M	HIV (CD4 41/μL)	Lymph node	Multiplex PCR	ATT (NR)/CLR	Improved
10	John et al., 2022 [43]	36/F	HIV (CD4 1/μL)/Parvovirus B19	Disseminated	PCR	ATT (HRZE)/CLR/	Improved
11	Sharma et al., 2012 [44]	2 Adults	HIV with meningitis (CD4 NR)	meninges	Multiplex PCR	NR	Both died
12	Lopez et al., 2025 [45]	45/M	Newly diagnosed HIV (low CD4)	Disseminated (lung/ extrapulmonary)	Culture/ molecular	ATT (HRZE)/AZM	Improved
13	Present case	49/F	HIV-negative, chronic inflammation (IGRA indeterminate)	Skin	mNGS/ serial cultures	ATT (INH/RPT/Lfx)/AZM/AMK/ topical RFP	Recovered

## Data Availability

All data presented in this report are included in the article. Further inquiries can be directed to the corresponding authors.

## Abbreviations

The following abbreviations are used in this manuscript:

AFB	Acid-fast bacilli
AMK	Amikacin
ATT	Anti-tuberculosis therapy
AZM	Azithromycin
CLR	Clarithromycin
CMV	Cytomegalovirus
CFX	Cefoxitin
CTB	Cutaneous tuberculosis
EMB	Ethambutol
HRZE	Isoniazid + rifampicin + pyrazinamide + ethambutol
IGRA	Interferon-γ release assay
INH	Isoniazid
Lfx	Levofloxacin
MAC	*Mycobacterium avium* complex
mNGS	Metagenomic next-generation sequencing
MTB	*Mycobacterium tuberculosis*
NTM	Nontuberculous mycobacteria
NR	Not reported
PZA	Pyrazinamide
RIF	Rifampicin
